# The hidden cost of connectivity: Social media addiction among medical students

**DOI:** 10.1192/j.eurpsy.2025.964

**Published:** 2025-08-26

**Authors:** A. Karmous, H. Ktari, H. Ghabi, A. Hajri, E. Khelifa, A. Maamri, H. Zalila

**Affiliations:** 1 Emergency and outpatient department, Razi hospital, Manouba; 2 Psychiatry department, Tahar Maamouri hospital, Nabeul; 3 Department G, Razi hospital, Manouba, Tunisia

## Abstract

**Introduction:**

Social media addiction (SMA) had not been formally recognized by medical organizations such as the American Psychiatric Association. However, it is an increasing problem especially among young adults. Little is known about medical students’ use of social media and the potential existence of this addiction among them.

**Objectives:**

The aim of the study was to investigate SMA among medical students and to determine the related factors.

**Methods:**

A cross-sectional study was conducted from February to March 2024 in the faculty medicine of Tunis (Tunisia). Full-time students, from the first to the fifth grade, who accepted to take part in the study were invited to respond to a questionnaire. Self-report questionnaires, comprising sociodemographic status, lifestyle habits, social media usage behavior, the Patient Health Questionnaire (PHQ-9), the Bergen Social media addiction scale (BSMAS) were filled by the participants.

**Results:**

A total of 60 individuals had fully responded to the questionnaire. The mean age was 21.98 ± 2.97 years. Eighty percent were female. Instagram was the most social media used with 96.6 % of participants followed by Facebook with 90 %. The mean number of hours spent on social media per day was 2.6 ± 1.96. According to the PHQ-9, 21.6 % (n=13) were suffering from depressive symptoms. The mean BSMAS was 16.6 ± 4.96 and 36.6 % (n=22) had SMA. SMA was more common among students in first years of medical school, not practicing a regular physical activity, posting regularly on social networks and having depressive symptoms.

**Conclusions:**

This study suggested that SMA is common among Tunisian medical students. An early intervention appears to be necessary to preserve their mental health and academic performance.

**Disclosure of Interest:**

None Declared

